# Inflammatory Findings on Musculoskeletal Ultrasound Do Not Affect Pain Intensity but May Influence Pain Quality in Patients with Refractory Arthralgia: A Cross-Sectional Study Using PainDETECT and Musculoskeletal Ultrasound

**DOI:** 10.7759/cureus.91169

**Published:** 2025-08-28

**Authors:** Mariko Harada, Michihiro Ogasawara, Shuko Nojiri, Yuko Matsuki-Muramoto, Toshio Kawamoto, Ken Yamaji, Naoto Tamura

**Affiliations:** 1 Internal Medicine and Rheumatology, Juntendo University School of Medicine, Tokyo, JPN; 2 Medical Technology Innovation Center, Juntendo University, Tokyo, JPN; 3 Clinical Research and Trial Center, Juntendo University, Tokyo, JPN

**Keywords:** diagnostic musculoskeletal ultrasound, neuropathic pain, paindetect questionnaire, refractory pain, rheumatoid arthriitis

## Abstract

Aim: The aim of this study was to investigate the prevalence and associated factors of neuropathic pain (NeP) in patients with refractory arthralgia and to evaluate the role of musculoskeletal ultrasound (MSKUS) in its assessment.

Materials and methods: A cross-sectional study was conducted in 187 patients with refractory arthralgia who underwent MSKUS to assess inflammation and NeP using the PainDETECT Questionnaire (PDQ). Clinical data, including demographics, disease activity, and functional status, were collected. The associations between NeP and clinical indicators, as well as MSKUS findings, were analyzed.

Results: A total of 187 patients were enrolled, of whom 41 (21.9%) were diagnosed with NeP. The strongest associated factors of pain in the overall cohort were PDQ score (p<0.001) and patient global assessment (PGA) (p<0.001). No significant correlation was found between pain and inflammatory findings on MSKUS. For the prediction of NeP, the numeric rating scale (NRS) score was the only significant associated factor (p<0.001, odds ratio (OR)=1.37, 95% confidence interval (CI): 1.17-1.60), whereas inflammation detected on MSKUS was not associated with NeP (p=0.19, OR=0.80, 95% CI: 0.57-1.12). Analysis of the PDQ components showed that burning pain severity was significantly higher in the moderate/high (M/H) inflammation group compared to the none/low (N/L) inflammation group (Mann-Whitney U test, p=0.032; logistic regression analysis, p=0.01, OR=1.53, 95% CI: 1.1-2.1).

Correlations between NRS and NeP were significant in both the N/L (Spearman's ρ=0.516, p<0.001; R²=0.2717, p<0.001) and M/H (Spearman's ρ=0.397, p<0.001; R²=0.1534, p<0.001) groups for all patients. However, within the rheumatoid arthritis (RA) subgroup, a significant correlation between NRS and NeP was observed only in the M/H group (Spearman's ρ=0.431, p=0.002; R²=0.212, p<0.001) but not in the N/L group (Spearman's ρ=0.251, p=0.3; R²=0.03849, p=0.4208).

Conclusion: NeP was most strongly associated with NRS and MSKUS. Inflammation may change the quality of pain rather than the intensity.

## Introduction

Rheumatoid arthritis (RA) is a chronic inflammatory disease characterised by synovitis and severe joint pain. Advances in RA management have allowed 50% to 64% of patients to live in remission or with low disease activity [1]. However, pain often persists despite proper treatment of inflammation based on the treat-to-target (T2T) strategy [2], and pain may increase over time after several years of treatment [3]. Non-inflammatory pain, such as neuropathic pain (NeP) and chronic widespread pain (CWP), has been identified as a cause of this persistent pain, in addition to nociceptive pain caused by synovitis [2,4,5]. In several studies of patients whose RA status ranged from remission to high disease activity, NeP was present in 14% to 40% of individuals [4,6-12], a percentage that was three times as high as that in the general population [6].

NeP is defined as “pain caused by a lesion or disease of the somatosensory system [13].” Patients with RA are known to have NeP-like pain, although there is often no peripheral or central nervous system lesion or disease. The following mechanism is supposed to be responsible: persistent pain or inflammation causes activation of joint nociceptors, peripheral sensitisation, and changes in central pain processing, resulting in increased central pain sensitisation and loss of descending analgesic mechanisms [14]. Although the mechanism is different, these RA symptoms are similar to those in NeP, including, for instance, burning pain and allodynia, and have been treated as NeP in many previous studies [7, 9, 11, 14, 15]. However, in this study, we will be discussing NeP, which has been the subject of many previous studies in relation to RA. NeP in RA contributes to physical dysfunction and decreased quality of life [7], so an early diagnosis and proper treatment are important. Non-inflammatory pain such as NeP is also a problem in systemic sclerosis (SSc), osteoarthritis (OA) [16], psoriatic arthritis (PsA), and Sjogren syndrome (SjS). Despite questionnaires used to identify NeP, such as the PainDETECT questionnaire (PDQ) [17], it may be challenging in daily medical practice to differentiate NeP from nociceptive pain solely based on clinical examination and blood tests.

NeP in RA is usually associated with patient-reported outcomes (PROs), especially those related to pain and psychiatric symptoms. Examples include health-related quality of life [16], visual analogue scale for pain (pain VAS), tender joint count (TJC) [4, 9], 36-item short form (SF36) mental component summary [9, 10], and SF36 physical component summary [10]. Body mass index (BMI) ≥22 kg/m^2^ and non-clinical remission [14] are also relevant. For non-RA, other factors have been reported, including the following: the Mean Modified Rodnan Skin Score [18] and the modified Medsger Severity Scale for muscle lesions in SSc; pain severity in OA [16]; small fibre neuropathy in SjS; and the swollen joint count (SJC), dactylitis, and fatigue in PsA. While blood test data indicating inflammation, such as C-reactive protein (CRP), were not found to correlate with NeP [4,14], in some cases, inflammation-related factors such as non-clinical remission and the presence of dactylitis have been found to predict NeP. Thus, the association between inflammation and NeP is still controversial.

Because musculoskeletal ultrasound (MSKUS) is the most sensitive means of detecting joint inflammation, patients with long-term or unexplained joint pain often undergo MSKUS. Even in clinical remission, the prevalence of power Doppler (PD) positivity in MSKUS was 44%, and this finding predicted the risk of relapse and structural progression after one to two years [19]. MSKUS can also detect early arthritis, and 89% of patients without joint inflammation were free of arthritis one year later [20]. The European Alliance of Associations for Rheumatology (EULAR) recommendations also suggest that MSKUS should be used to determine the presence of inflammation in difficult-to-treat RA (D2TRA) [21]; if MSKUS shows no joint inflammation, synovitis can be ruled out, and non-pharmacologic therapy is recommended. However, pain quality in patients who are PD negative has not been investigated.

For this reason, patients with refractory arthralgia visit the MSKUS outpatient department. The aim of this study was to explore the factors most strongly associated with pain and to investigate the prevalence and associated factors of NeP, and to examine the role of MSKUS in the assessment of NeP using MSKUS and the PDQ in this group of patients with refractory arthralgia visiting the MSKUS outpatient department.

## Materials and methods

Study design and population

This observational, cross-sectional study was conducted at the Department of Internal Medicine and Rheumatology, Juntendo University School of Medicine in Tokyo, Japan. From August 2020 to June 2021, we recruited consecutive patients with refractory arthralgia who underwent MSKUS to evaluate inflammation and conducted the PDQ to evaluate the presence of NeP. Patients with known causes of neuropathy, for example, spinal canal stenosis, carpal tunnel syndrome, diabetic neuropathy, and cervical hernia, were excluded from the study. The ethics committee at Juntendo University approved this study (study no. H21-0021). All participants gave written informed consent according to the Declaration of Helsinki.

Variables and outcome measures

Clinical Data

The following patient data were collected from medical records: age, gender, diagnosis, disease duration, past history, medication history (prednisolone (PSL), methotrexate (MTX), biologic synthetic disease-modifying anti-rheumatic drugs (bDMARDs), targeted synthetic disease-modifying anti-rheumatic drugs (tsDMARDs), nonsteroidal anti-inflammatory drugs (NSAIDs), and anti-anxiety drugs), and blood tests (rheumatoid factor (RF), anti-cyclic citrullinated peptide antibody (ACPA), anti-SS-A antibody, CRP, and erythrocyte sedimentation rate (ESR)).

Disease Activity Evaluation

RA disease activity was evaluated by the disease activity score for 28 joints (DAS28)-ESR and DAS28-CRP. Together, these include SJC and TJC for 28 joints, as well as a patient global assessment (PGA) for general disease activity (0-10) based on questionnaire responses, and CRP and ESR levels. The NRS score was also evaluated using a questionnaire (0-10). In non-RA arthritis, disease activity was not evaluated.

Functional Evaluation

Functional disability was assessed by the Health Assessment Questionnaire Disability Index (HAQ). On a questionnaire comprising items rated on a four-point scale, patients indicated how difficult it was to perform 20 daily activities.

PDQ

We evaluated NeP using the Japanese version of the PDQ [22]. Although the definition of neuropathic pain in the narrow sense requires pain and sensory abnormalities consistent with the site of innervation, the accuracy of the PDQ as a screening tool is well accepted. The PDQ has a sensitivity of 85% and a specificity of 80% in differentiating nociceptive pain from NeP [23]. It was also indicated to be useful for the evaluation of NeP in RA [11], OA, fibromyalgia, and diverse musculoskeletal conditions [23]. The PDQ score consists of the pattern of pain over the course of the disease, the presence of radiating pain, gradation of pain, and quality of pain (burning, tingling or prickling, allodynia, sudden pain attack, hypersensitivity to cold or heat, numbness, or hypersensitivity to pressure). The total score ranges from 0 to 38 points. Scores ≤12 indicate unlikely NeP, scores ≥19 indicate likely NeP, and values from 13 to 18 indicate possible NeP. Based on the above, we classified patients with a score ≤12 as the non-NeP group and those with a score ≥13 as the NeP group [4,7,10,11,14]. Additionally, this questionnaire simultaneously assessed the intensity of pain, including the current NRS, the severity of the most intense pain experienced in the past, and the average pain level over the past four weeks.

Radiographic Damage

X-rays of the hand or wrist taken within one year from the date of MSKUS were categorised using the Steinbrocker classification into four stages by a single examiner who was blind to patient clinical data, diagnosis, and medication.

MSKUS

MSKUS was performed using an ARIETTA 70 US unit (Fujifilm Healthcare Corporation, Tokyo, Japan) equipped with a high-frequency linear transducer L64 (5-18 MHz). MSKUS was performed by three rheumatologist sonographers (MO, TK, and YM), each with more than five years of experience in MSKUS (more than 2,500 cases) and blind to patient diagnosis, clinical data, and radiographic data.

We conducted MSKUS examination of all painful joints and examined the wrist, interphalangeal (IP) joint, proximal interphalangeal (PIP) joint, and metacarpophalangeal (MCP) joint bilaterally. Longitudinal and transverse scans were performed using both B-mode and Doppler mode. Synovial thickening and effusion were defined as hypoechoic areas of the joint, tendon, and bursa and were semiquantitatively graded using the four-point Grey Scale, with scores ranging from 0 to three. PD signals were used to display flow signals in the synovium and were semiquantitatively graded using the four-point PD scale, with scores ranging from 0 to three [24]. For all test results, the maximum grade was taken as the total MSKUS score of the patient, which was classified into four levels: no, low, moderate, or high.

All grade decisions were made by MO, who has completed the EULAR Sonography course (Advanced Musculoskeletal Ultrasound in Rheumatology and Course for Ultrasound Trainers in Rheumatology: Teach the Teachers). Factors that can influence US findings, such as NSAID use, room temperature, and body position, were considered.

Statistical methods

To assess the contributing factors to pain, multiple regression analysis was used to examine patient background, blood and imaging test results, and medication history for the whole patient group and for the group of RA patients. And, to determine the associated factors of NeP, we compared the NeP and non-NeP groups. Data were presented as mean (SD) for continuous variables and as frequencies for categorical variables. Univariate analysis was performed using Fisher’s exact test for normally distributed variables and the Mann-Whitney U (MWU) test for non-normally distributed variables. Multivariate analysis was performed using logistic regression, and an age-adjusted model was created that included US findings of interest and items that were significantly different (P<0.05) in the univariate analysis. The multiple imputation analysis included all variables used in the final analysis with 10 imputations by using the multivariate imputation by chained equations (MICE) [25] method, applying PMM to continuous variables (SS-A, CCP, RF, CRP, ESR) and POLR to categorical variables (XP stage) to produce imputed data. We confirmed that there was no significant difference between 10 and 100 iterations in the missing value imputation method. We conducted analyses with two settings, m=10 and m=100, and confirmed the consistency of the results. When comparing the imputation results for m=10 and m=100, all variables showed high consistency in terms of mean and median values.

To investigate the association of inflammation and quality of pain, patients were divided into 2 subgroups based on the presence of no/low US inflammation (N/L group) or moderate/high US inflammation (M/H group). The MWU test was performed on PDQ components between the N/L and M/H groups as a univariate analysis, followed by logistic regression analysis.

To further investigate the association between NeP and US-determined no/low inflammatory findings, divided into N/L and M/H groups, a subgroup analysis was then conducted. In each group, Spearman's rank correlation coefficient between NRS and NeP was determined, and single regression analyses were performed. Next, we conducted the same test focusing on the RA patient group to investigate the correlation between NeP and NRS scores. In addition, a comparison between the N/L group and the M/H group was performed using simple regression analysis in the RA group. Receiver operating characteristic (ROC) curve analysis was also conducted on the RA patient group to calculate the cut-off value of the NRS score that can diagnose NeP.

Statistical significance was defined as p≤0.05. All analyses were performed with EZR (V.1.54; Saitama Medical Center, Jichi Medical University, Saitama, Japan) [26] and SAS (V.9.4; SAS Inc., Cary, NC).

## Results

Patient characteristics

**Figure 1:**
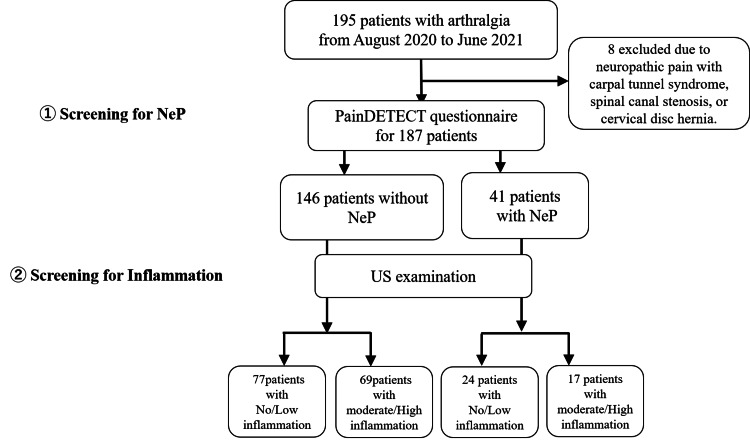
Study flowchart Patients with arthralgia who were examined by US at our department and interviewed with the PDQ to evaluate the presence of NeP were enrolled in this study from August 2020 to June 2021, excluding patients with diagnosed neuropathy. PDQ: painDETECT questionnaire; NeP: neuropathic pain; US: ultrasound

In total, 195 patients underwent MSKUS during the study period, and 187 were enrolled after eight were excluded because NeP apparently had causes other than arthralgia (carpal tunnel syndrome, three; spinal canal stenosis, two; cervical disc hernia, three) (Figure 1).

Of the 187 patients, the median (range) age was 55 (16-83) years, and 148 (79.1%) patients were females. Seventy patients (37.4%) had RA, 80 (42.8%) had undiagnosed arthralgia, and the remaining 37 had other rheumatic diseases (SjS, eight; polymyalgia rheumatica, eight; PsA, five; mixed connective tissue disease, four; systemic lupus erythematosus, two; gout, two; remitting seronegative symmetrical synovitis with pitting edema, two; ulcerative colitis, one; SSc, one; familial Mediterranean fever, one; polymyositis, one; polyarteritis nodosa, one; Takayasu arteritis, one; antiphospholipid syndrome, one; SAPHO (synovitis, acne, pustulosis, hyperostosis and osteitis) syndrome, one; and Behçet's disease, one). Forty-one patients (21.9%) had NeP (Table 1).

Associated factors of pain

The strongest correlations with pain were PDQ score (p<0.001) and PGA (p<0.001) in the overall group, and in the RA group, the strongest correlations were PDQ score (p=0.04) and PGA (p<0.001). There was no significant correlation in inflammatory findings on MSKUS with X-ray findings, blood test results, or medications.

Associated factors of NeP

The NeP group had a significantly higher NRS score (p<0.001), PGA (p=0.005), TJC (p=0.001), SJC (p=0.001), and HAQ score (p=0.002) than the non-NeP group. There were no significant differences in inflammatory findings on MSKUS, X-ray findings, blood test results, or medications (Table 1).

**Table 1:** Characteristics of patients with unlikely NeP versus likely and possible NeP NeP: neuropathic pain; RA: rheumatoid arthritis; NRS: numerical rating scale score; Average: average pain in the last week; Greatest: greatest pain in the last week; PGA: patient global assessment; TJC: tender joint count; SJC: swollen joint count; HAQ: health assessment questionnaire; US: ultrasound; MTX: methotrexate; DMARD: disease-modifying anti-rheumatic drug; PSL: prednisolone; NSAID: non-steroidal anti-inflammatory drug; CRP: c-reactive protein; ESR: erythrocyte sedimentation rate; ACPA: anti-cyclic citrullinated peptide antibody; RF: rheumatoid factor; Other diagnoses: Gout, Sjögren's syndrome, psoriatic arthritis, polymyalgia rheumatica, systemic lupus erythematosus, RS3PE syndrome, systemic sclerosis, mixed connective tissue disease, ulcerative colitis, familial Mediterranean fever, dermatomyositis, Behçet's disease, SAPHO (synovitis, acne, pustulosis, hyperostosis and osteitis) syndrome, Takayasu arteritis, osteoarthritis, anti-phospholipid antibody syndrome *Fisher’s exact test; *Mann–Whitney U test

Variable	Overall	Unlikely NeP	Likely and possible NeP	p-value
n		146	41	
Age (years) – median (min-max)	55 (16–83)	55 (16–83)	57 (17–78)	0.28**
Female sex – n (%)	148 (79)	114 (78)	34 (83)	0.66*
Diagnosis				
RA, n (%)	70 (37)	58 (40)	12 (29)	0.5*
Not diagnosed, n (%)	80 (43)	60 (41)	20 (49)	
Others, n (%)	37 (20)	28 (19)	9 (22)	
Symptoms duration (years), median (min-max)	2 (0–33)	2 (0–33)	2 (0–22)	0.83**
NRS score, median (min-max)	4 (0–10)	4 (0–10)	7 (0–9)	<0.001**
Average, median (min-max)	5 (0–10)	4 (0–9)	7 (1–10)	<0.001**
Greatest, median (min-max)	7 (0–10)	5 (0–10)	9 (4–10)	<0.001**
PGA, median (min-max)	5 (0–10)	4 (0–10)	5 (0–10)	0.005**
TJC53, median (min-max)	6 (0–53)	5 (0–53)	11 (1–36)	0.001**
SJC, median (min-max)	1 (0–43)	0 (0–43)	3 (0–42)	0.001**
HAQ, median (min-max)	0.25 (0–2.8)	0.12 (0–2.8)	0.38 (0–2.5)	0.002**
US inflammation, n (%)				0.88*
No	69 (37)	53 (36)	16 (39)	
Low	32 (17)	24 (16)	8 (20)	
Moderate	51 (27)	40 (27)	11 (27)	
High	35 (19)	29 (20)	6 (15)	
X-ray Steinblocker grade, n (%)				
1	137 (73)	103 (71)	34 (83)	0.29*
2	29 (16)	26 (18)	3 (7)	
3	14 (8)	12 (8)	2 (5)	
4	7 (4)	5 (3)	2 (5)	
Mental disorder, n (%)	18 (10)	12 (8)	6 (15)	0.24*
Antidepressant, n (%)	18 (10)	12 (8)	6 (15)	0.24*
MTX (mg/week), median (min-max)	0 (0–14)	0 (0–14)	0 (0–12)	0.57**
MTX (previous/current), n (%)	58 (31)	49 (34)	9 (22)	0.18*
MTX duration (month), median (min-max)	0 (0–228)	0 (0–228)	0 (0–134)	0.13**
csDMARDs, n (%)	37 (20)	31 (21)	6 (15)	0.51*
bDMARDs, n (%)	14 (8)	13 (9)	1 (2)	0.31*
bDMARDs (previous/current), n (%)	20 (11)	19 (13.0)	1 (2)	0.082*
bDMARDs duration (month), median (min-max)	0 (0–240)	0 (0–240)	0 (0–13)	0.051**
PSL (mg), median (min-max)	0 (0–30)	0 (0–30)	0 (0–30)	0.15**
PSL (previous/current), n (%)	47 (25)	35 (24)	12 (29)	0.54*
PSL duration (months), median (min-max)	0 (0–468)	0 (0–468)	0 (0–264)	0.63**
NSAIDs, n (%)	81 (43)	62 (43)	19 (46)	0.72*
Pregabalin, n (%)	10 (5)	6 (4)	4 (10)	0.23*
CRP (mg/dL), median (min-max)	0.12 (0–7.6)	0.14 (0–7.6)	0.09 (0–5.4)	0.46**
ESR (mm), median (min-max)	11 (2–124)	11 (2–124)	11 (2–53)	0.55**
ACPA (U/mL), median (min-max)	0 (0–1250)	0 (0–1250)	0 (0–394)	0.064**
RF (IU/mL), median (min-max)	13.1 (0–1050)	12.5 (0–1050)	16.1 (0–446.9)	0.95**
SS-A (U/mL), median (min-max)	0 (0-128)	0 (0-128)	0 (0-32)	0.43**

In logistic regression analysis, only the NRS score was significantly associated with NeP (p<0.001, odds ratio (OR)=1.37, 95% CI: 1.17-1.60); US inflammation showed no association with NeP (p=0.19, OR=0.80, 95% CI: 0.57-1.12) (Table 2).

**Table 2:** Logistic regression associated with NeP Logistic regression analysis was performed for items with significant differences in Table 1 and for US inflammation, after adjustment for age. NeP: neuropathic pain; NRS: numerical rating scale score; US: ultrasound; SJC: swollen joint count; OR: odds ratio

Variable	OR (95% CI)	P-value
NRS	1.37 (1.17–1.60)	<0.001
US inflammation	0.80 (0.57–1.12)	0.19
Age	1.01 (0.98–1.04)	0.50
SJC	1.03 (0.98–1.08)	0.27

Differences in PDQ components with and without US inflammation

The severity of burning pain was significantly higher in the M/H group than in the N/L group by the MWU test (p=0.032) (Table 3) and in logistic analysis (p=0.01, OR=1.53, 95% CI: 1.1-2.1).

**Table 3:** PainDETECT questionnaire items: N/L group vs M/H group N/L group: patients with no/low synovitis; M/H group: patients with moderate/high synovitis; VAS: visual analog scale; Mann–Whitney U test was used.

Variable	Overall	N/L group	M/H group	p-value
n	187	99	88	
Pain course pattern - n (%)				0.635
Persistent pain with slight fluctuations	83 (44)	43 (43)	40 (46)	
Persistent pain with pain attacks	34 (18.2)	16 (16)	18 (21)	
Pain attacks without intervening pain	48 (26)	29 (29)	19 (22)	
Pain attacks with intervening pain	22 (12)	11 (11)	11 (13)	
Radiating pain - n (%)	31 (17)	15 (15)	16 (18)	0.694
Pain quality- median (min-max)				
Burning	0 (0–5)	0 (0–4)	1 (0–5)	0.032
Tingling or prickling	1 (0–5)	1 (0–5)	1 (0–4)	0.723
Allodynia	1 (0–5)	1 (0–5)	1 (0–4)	0.749
Sudden pain attacks/electric shocks	1 (0–5)	1 (0–5)	1 (0–4)	0.588
Hypersensitivity to cold or heat	0 (0–5)	0 (0–5)	0 (0–3)	0.411
Numbness	1 (0–5)	1 (0–5)	1 (0–4)	0.232
Hypersensitivity to pressure	2 (0–5)	2 (0–5)	2 (0–5)	0.541
Pain VAS (cm) - median (min-max)	4 (0–10)	4 (0–9)	4 (0–10)	0.452
Mean (cm) - median (min-max)	5 (0–10)	5 (0–10)	5 (0–10)	0.56
Strong (cm) - median (min-max)	7 (0–10)	7 (0–10)	6 (0–10)	0.724

Association between US inflammation, NeP, and pain

Correlation Between Pain and PainDETECT With and Without US Inflammation

Although the overall analysis in Table 1 showed no significant association between NeP and US inflammation, we predicted an association between NRS, NeP, and US inflammation, so we performed a subgroup analysis by dividing into the N/L and M/H groups.

Correlations between NRS and NeP were observed in both the N/L (Spearman’s ρ=0.516, p<0.001; R²=0.2717, p<0.001) and M/H groups (Spearman’s ρ=0.397, p<0.001; R²=0.1534, p<0.001) in entire patients. However, in the analysis of only RA patients, NRS was significantly correlated with NeP only in the M/H group (Spearman’s ρ=0.431, p=0.002; R²=0.212, p<0.001) but not in the N/L group (Spearman’s ρ=0.251, p=0.3; R²=0.03849, p=0.4208).

In the RA group, a comparison using simple regression analysis between the N/L group and the M/H group showed that ESR (p=0.001), CRP (p=0.002), ACPA (p=0.004), duration of bDMARDs (p=0.013), history of bDMARDs (p=0.03), RF (p=0.037), and antidepressant use (p=0.04) were higher in the M/H group (Table 4). There was no significant difference in NRS and PDQ scores.

**Table 4:** Characteristics of patients with N/L and M/H in the RA group RA: rheumatoid arthritis; N/L group: no/low US inflammation; M/H group:  moderate/high US inflammation; PGA: patient global assessment; TJC: tender joint count; SJC: swollen joint count; HAQ: health assessment questionnaire; US: ultrasonography; MTX: methotrexate; DMARD: Disease modifying anti rheumatic drug; PSL: prednisolone; NSAIDs: non-steroidal anti-inflammatory drugs; CRP: C-reactive protein; ESR: erythrocyte sedimentation rate; ACPA: anti-cyclic citrullinated peptide antibody; RF: rheumatoid factor

Variable	N/Lgroup	M/Hgroup	p-value
n	19	51	
Age (years) – median (min-max)	52 (22-77)	57 (18-83)	0.26
Female sex, n (%)	15 (78.9)	44 (86.3)	0.47
Symptoms duration (years), median (min-max)	4 (1-22)	6 (0-33)	0.84
NRS score, median (min-max)	4(1-9)	4 (0-9)	0.34
Average, median (min-max)	5 (2-9)	5 (0-10)	0.62
Greatest, median (min-max)	7 (2-10)	6 (0-10)	0.53
PainDETECT, median (min-max)	8 (2-14)	6 (0-19)	0.21
PGA, median (min-max)	4 (1-10)	5 (0-9)	0.86
TJC53, median (min-max)	11 (0-53)	5 (0-32)	0.14
SJC, median (min-max)	1 (0-17)	2 (0-20)	0.39
HAQ, median (min-max)	0.12 (0-1.88)	0.38 (0-2.25)	0.14
X-ray Steinblocker grade, n (%)			
1	9 (47.4)	19 (37.3)	0.59
2	4 (21.1)	19 (37.3)	
3	4 (21.1)	8 (15.7)	
4	2 (10.5)	5 (9.8)	
Mental disorder, n (%)	2 ( 10.5)	0 (0)	0.07
Antidepressant, n (%)	4 (21.1)	2 (3.9)	0.04
MTX (mg/week), median (min-max)	0 (0-12)	0 (0-12)	0.78
MTX (previous/current), n (%)	12 (63.2)	29 (56.9)	0.79
MTX duration (month), median (min-max)	4 (0-228)	4 (0-160)	0.84
csDMARDs, n (%)	11 (57.9)	11 (37.3)	0.18
bDMARDs, n (%)	6 (31.6)	5 (9.8)	0.06
bDMARDs (previous/current), n (%)	7 (36.8)	6 (11.8)	0.03
bDMARDs duration (month), median (min-max)	0 (0-240)	0 (0-12)	0.013
PSLmg	0 (0-5)	0 (0-10)	0.63
PSL (previous/current), n (%)	7 (36.8)	18 (35.3)	1
PSL duration (months), median (min-max)	0 (0-92)	0 (0-468)	0.78
NSAIDs, n (%)	9 (47.4)	24 (47.1)	1
Pregabalin, n (%)	2 (10.5)	0 (0)	0.07
CRP (mg/dL), median (min-max)	0.04 (0-0.56)	0.21	0.002
ESR (mm), median (min-max)	7 (2-41)	17 (2-91)	0.001
ACPA (U/mL), median (min-max)	0 (0-300)	73.1 (0-1250)	0.004
RF (IU/mL), median (min-max)	11.4 (0-143.8)	33.7 (0-446.9)	0.037
SS-A (U/mL), median (min-max)	0 (0)	0 (0-32)	0.03

ROC Curve

**Figure 2:**
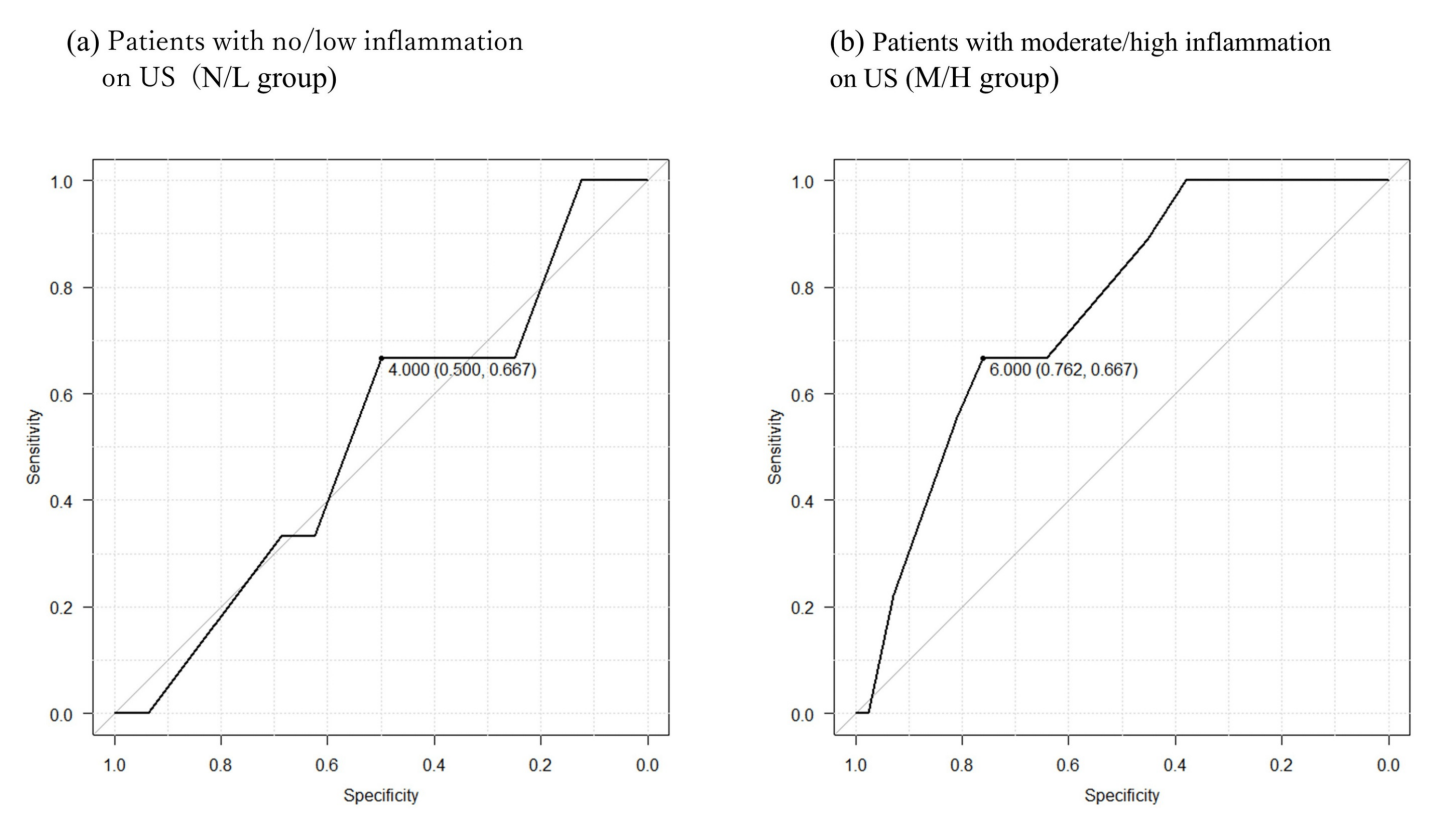
The optimal NRS cut-off score for diagnosing NeP in the RA group In the RA group, the ROC curve analysis was performed to determine the NRS cut-off for diagnosing NeP in both N/L and M/H groups. (a) N/L group. Cut-off: 4, 50% sensitivity and 66.7% specificity, AUC=0.521, 95% CI 0.132-0.91; (b) M/H group. Cut-off: 6, 76.2% sensitivity and 66.7% specificity, AUC=0.757, 95% CI 0.601-0.912. ROC: receiver operating characteristic; NRS: numeric rating scale; RA: rheumatoid arthritis; N/L group: patients with no/low synovitis; M/H group: patients with moderate/high synovitis; US: ultrasound; AUC: area under the curve

To investigate the contribution of NeP to NRS in the presence or absence of US inflammation in the RA group, ROC curve analysis was used to determine the NRS cut-off that determined the presence of NeP. In the N/L group, the optimal cut-off was four (50% sensitivity and 66.7% specificity, area under the curve (AUC)=0.521, 95% CI 0.132-0.91), while in the M/H group, the optimal cut-off was six (76.2% sensitivity and 66.7% specificity, AUC=0.757, 95% CI 0.601-0.912) (Figure 2). The ROC curve for the N/L group had a lower AUC (0.521) than the M/H group (0.757), and was less reliable.

## Discussion

In this study, we conducted a multi-faceted analysis of refractory pain of unknown origin using the PDQ to assess non-inflammatory pain and MSKUS to assess the presence or absence of inflammation. The results showed that pain intensity was significantly associated with non-inflammatory pain, but there was no association with inflammatory findings detected by MSKUS. It was also found that non-inflammatory pain could not be predicted from patient background factors and that only pain intensity was a useful associated factor. In addition, it was suggested that inflammatory findings detected by MSKUS were related to pain quality as burning pain, and may have a greater impact on pain quality than pain intensity. These results clearly distinguish the effects of inflammatory and non-inflammatory factors in refractory pain and suggest the need for more personalized pain management.

In patients with unexplained chronic joint pain, non-inflammatory factors such as NeP and PGA were more associated with pain than inflammatory factors. NeP was significantly associated with pain, as previously reported [4, 11, 15]. Pain should not be underestimated, even in the absence of US inflammation. A previous study showed a discrepancy between PGA and Evaluator Global Assessment (EGA); specifically, the main determinant of PGA was pain, and the determinant of EGA was SJC [27]. This suggests that physicians were not focusing enough on pain, and this issue should be addressed in relation to NeP.

The quality of pain in this trial varied according to the presence or absence of US inflammation. Burning pain was more severe in patients with US inflammation. Burning pain was the most common NeP symptom and was also common in patients with RA [28]. It was associated with damage to the nociceptive system [29]. In a review of post-herpetic neuralgia, burning pain was common in herpes zoster infection due to sensitization of cutaneous nociceptor endings (irritable nociceptors) by the herpes rash and inflammation [30]. The finding that burning pain was more severe in patients with MSKUS inflammation suggests the presence of peripheral sensitization due to inflammation. Thus, even in patients with high PDQ scores, the mechanism of pain may differ depending on the presence or absence of US inflammation: a high PDQ score in patients with US inflammation may indicate peripheral sensitization induced by inflammation, whereas a high PDQ score in patients without US inflammation may be due to true NeP without inflammation. The combined use of US and PDQ may accurately identify NeP. Therefore, if the US is negative for inflammation, analgesic therapy should be considered rather than increasing DMARDs.

In the RA group, the presence or absence of US inflammation was associated only with inflammatory factors such as CRP and RF, and there was no association with pain intensity or the presence or absence of NeP, as previously. However, the significant correlation between NRS and NeP was observed both with and without US inflammation in all patients, but in RA patients, the correlation was only observed in the group of RA patients with US inflammation. When RA patients have inflammation, the symptoms may be NeP-like due to the quality of pain, with burning pain. Inflammation may change the quality of pain rather than the intensity of pain. When using the PDQ in RA patients, it may be necessary to be aware that burning pain increases in the group of patients with high MSKUS inflammation, which may lead to more patients being considered NeP. The cause of pain in RA without inflammation may be complex and not only explained by NeP.

Patients without MSKUS inflammation but with a high NRS should be treated for potential NeP, whereas patients with MSKUS inflammation may have peripheral nociceptor sensitization due to inflammation and may benefit from anti-inflammatory drug treatment. This is consistent with the algorithm of the EULAR recommendation [21] that MSKUS should be performed for D2TRA and that anti-inflammatory drug therapy should be considered if US inflammation is present, whereas non-pharmacological therapy (education, exercise, self-management) is preferable to increasing the dose of DMARDs if US inflammation is absent.

There are several limitations to this study. Because the participants included not only people with RA but also people with undiagnosed joint pain and other rheumatic diseases, not all of the results may be applicable to RA. In addition, because MSKUS is indicated when the diagnosis or cause of joint pain is unknown, patients who undergo MSKUS are more likely to complain of pain, which introduces a selection bias. Also, although mental illness was not associated with NeP in this study, the results may have been different if current mental status had been assessed using the SF36 or other methods, as the study analyses were based on medical record data only. Recently, NeP in RA has been reported to be part of a central sensitization syndrome [31], and the Central Sensitization Inventory (CSI) has also been used as a questionnaire. Future surveys using the CSI are being considered.

A strength of this study is the combined use of the PDQ to evaluate non-inflammatory pain and MSKUS to objectively detect inflammatory changes. This enabled a clear distinction between inflammatory and non-inflammatory components of refractory joint pain and how pain intensity and quality are influenced by inflammation. While EULAR recommendations have noted the importance of the presence or absence of US inflammation in the treatment of refractory RA, this study introduces a novel finding by identifying that the quality of pain may differ depending on the presence or absence of US inflammation. This finding further supports the recommendations. Clinically, these findings may contribute to more personalized and effective management of refractory joint pain. 

## Conclusions

We conducted an observational, cross-sectional study of patients with unexplained arthralgia, using US and PDQ. The results showed that inflammatory findings detected by MSKUS were related to quality of pain, such as burning pain, and may have a greater impact on pain quality than pain intensity. It may contribute to more personalized and effective management of refractory joint pain.
